# Proteid Poisons of Jequirity

**Published:** 1889-10-19

**Authors:** 


### RECENT RESEARCH.

### PROTEID POISONS OF JEQUIRITY.

Dr. Sidney Martin, British Medical Association Research Scholar, has forwarded an interesting report on "Proteid Poisons, with Special Reference to that of the Jequirity (*abrus precatorius*)."* The list of "proteid poisons" is extending, and is divisible into two groups—viz., animal and vegetable proteid poisons. Among animal proteid poisons is, first and foremost, the venom of snakes; next, the serum of the conger eel; thirdly, poisons formed during digestion, *albumoses* and *peptones*; fourthly, proteid poisons of certain spiders. "To these may be added the body, which produces coagulation of the blood *intra vitam*." Of vegetable proteid poisons, there are jequirity, papam, lupinotoxin. The seeds of jequirity contain a substance which, when extracted as a watery infusion, produces local inflammation of the conjunctiva, and this has been utilised practically for the cure of granular lids and of pannus. From researches of continental observers, it was concluded that the irritant action of jequirity was due to a special bacillus which grew in the infusion of the seeds. But Dr. Martin disposes of this idea by stating that he has found no bacteria present. We must look, therefore, for a proteid, two of which Dr. Martin found—viz., a globulin and an albumose. The author next details how these proteids are separated, and he states that both these proteids possess the property of causing inflammation of the conjunctiva, but the globulin is more poisonous than the albumose, and more likely to act injuriously on the general system. The early symptom of poisoning is fall of temperature, and this may be followed by gastro-enteritis The author further considers that there is a strong relation between these proteids and the poison of snake venom, although they are not identical. At present it is not explainable why proteids should be poisonous. Indeed, we do not know whether a proteid is of itself poisonous, or possesses toxic properties by virtue of some agent formed from it. Such an agent would be called a ferment. The fact which would point to abrus poison being a ferment is, that its activity is permanently destroyed by a moist heat below 100deg. C. Further than this, we cannot at present even surmise.

### ANTISEPTICS.

A paper by C. T. Kingzett, F.I.C., F.C.S., "On the Comparative Antiseptic Value of Chlorides, Nitrates, Sulphates, and other Chemical Substances,"† demands atten-

* *British Medical Journal*, July 27, 1889.

